# General Medical Council, Great Britain—Abstract of Discussion upon Materia Medica

**Published:** 1898-10

**Authors:** 


### GENERAL MEDICAL COUNCIL, GREAT BRITAIN—ABSTRACT OF DISCUSSION UPON MATERIA MEDICA.

*Dr. Heron Watson.*—I have to note that it is not taken notice of that materia medica has been entirely dropped out. I am quite aware there are some remarks made, explaining that materia medica is supposed to be included in "dental surgery, pathology, and therapeutics;" further on in the recommendations, that "therapeutics" are presumed to cover the whole. I merely mention this in order that I shall not be told that all this has been passed, and that I had no right to speak later on. I have no objection to deferring any remarks I have to make.

*Mr. Bryant.*—I beg to move "that recommendations (v.) and (v.) (*a*) read as follows: '(v.) That he has attended courses of instruction in the following special subjects at a recognized dental school: (*a*) dental anatomy and physiology, human and comparative, with practical work and demonstrations in dental histology for three months.'"

*Mr. Tomes.*—I beg to second that.

*Mr. Tichborne.*—What is a recognized dental school?

*Dr. MacAlister.*—A dental school recognized by one of the licensing bodies.

*Mr. Tichborne.*—A medical school?

*The President.*—If there is a dental department attached to the school, I presume.

*Mr. Carter.*—Would it not be as well to introduce the words "for at least the period specified" after the words "dental school."

*Mr. Bryant.*—Yes, there is not the least objection to that; I will put that in my proposal.

The resolution was put to the Council as follows: "(v.) That he has attended courses of instruction in the following special subjects, at a recognized dental school, for at least the period specified: (*a*) dental anatomy and physiology, human and comparative, with practical work and demonstrations in dental histology for three months."

This was agreed to.

*Mr. Bryant.*—I beg to move "that recommendation (v.) (*b*) read as follows: '(*b*) Dental surgery and pathology for three months, including materia medica and therapeutics applicable to dental surgery.'" Perhaps, anticipating objections which may be raised, I may say that we thought it was expedient to cut out the whole course of lectures on materia medica, and really the student was not wanted to know the whole materia medica, but only as much as is applicable to his own branch of the profession, and that therefore it was as well to remove that load from his shoulders. In a certain institution, which shall be nameless, that has been cut out. There is not the least reason why the student should not be called upon to attend the six months' course in materia medica if he likes, with six lectures a week. The committee think that the student is fairly well loaded already, and that we may fairly cut that materia medica out. The same institution, which shall be nameless, has cut it out from the general medical students.

*Mr. Tomes.*—I beg to second the recommendation.

*Dr. Leech.*—I am quite in favor of the materia medica as a whole course being struck out, but I hardly like the way in which it is put here. I know the lectures with us are given separately. Therefore this does not read well. I think it would be better to read "together with a short course of materia medica applicable to dental surgery."

*Mr. Bryant.*—I do not think that was the intention of the committee. It leaves it open for an extra course of materia medica if a school wishes it. It was thought probable and advisable that sufficient teaching in the materia medica would come in the treatment of dental surgery in the affections which come under the notice of the dental surgeon. But the treatment of those would be dealt with by the lecturer. That is merely to satisfy the requirements of some of the schools. Others may give a short course, and others may think it better to let the student have a complete course of materia medica. Any one of those three methods are open to the teaching bodies, and therefore it was thought that it should be introduced as I have read it to you.

*Dr. Pettigrew.*—I think it is quite obvious that the materia medica does not naturally fall either under dental surgery or pathology. I think it ought to be separated, as indicated by Dr. Leech.

*Sir C. Nixon.*—Why a dental student should have a knowledge of the materia medica I really cannot understand.

*Dr. MacAlister.*—It is only so much of it as is applicable to dental surgery. Dental surgery includes the treatment of the teeth, and may include mouth-washes and antiseptics, and so on.

*Sir Wm. Gairdner.*—It would be better if the dentists had short courses of these things suitable for themselves.

*Dr. Leech.*—Surely the dentist should be familiar with the therapeutics of arsenic. Again, he is using carbolic acid constantly. He should know something of the things which he uses. I think a short course of a few lectures would be enough for him. It seems to me it would be better to mention that. I am very glad that materia medica is cut out, but a man should know something about the things which he is employing.

*Mr. Bryant.*—Do you not think that the resolution is sufficient?

*Dr. Leech.*—It seems so little that it will practically lapse altogether.

*Mr. Bryant.*—We will put "with" if you like, instead of "including."

*Mr. Carter.*—Although partly responsible for the report, I do not see how the surgery could include materia medica.

*Mr. Bryant.*—Put the word "with."

*Mr. George Brown.*—Perhaps Mr. Tomes could throw some light on this subject, as to the necessity of a thorough knowledge of materia medica. I remember a book entitled "Dental Materia Medica," which appeared to me to contain a very extensive range, not only in the preparation of mouth-washes and stoppings for the teeth, but also as to the administration of drugs. I should like to know whether that is the case or not.

*Dr. Watson.*—It is obviously the intention of the committee that they should not only condemn materia medica with faint praise, but that they should really eliminate it altogether. No doubt if the course of dental surgery and pathology contains any requisite on this subject, a student may be the better for it, and a conscientious teacher, who wishes to make up for want of interest on the part of this Council in connection with that subject, may feel it his duty to extend it to his course. The part to which I previously referred, namely, of "Dental surgery, pathology, and therapeutics," seems extremely bare when taken by itself; you will find there is no mention there of materia medica. Does that include materia medica or does it not? I think we are accepting a serious responsibility in doing this. I am confident that however little we may wish dentists to write prescriptions, that they do such things, and that there are not a few students who will become dentists and carry on at the same time a connection with this business at a profit. Under those circumstances, they should be called upon to have a competent knowledge of what may be deemed necessary for a patient coming under their care.

*Mr. Bryant.*—That is rather an assumption on the part of Dr. Watson. A student will be taught, we hope, and will be examined in materia medica and therapeutics so far as it is applicable to the department of surgery, and no more.

*Dr. Heron Watson.*—In the professional examinations there is not one word about materia medica.

*Mr. Bryant.*—We do not wish to encourage dentists to prescribe for ordinary diseases.

*The President.*—There has been put into my hands by Mr. Bryant a slight modification of the phraseology of "(*b*) Dental pathology and surgery with materia medica and therapeutics in their application to dental surgery for three months."

The resolution as amended by Mr. Bryant was agreed to.—*Journal of the British Dental Association.*

